# Satisfaction of Using Continuous Glucose Monitoring for Care Partner‐Assisted Diabetes Management for Older Chinese Americans with Mild ADRD

**DOI:** 10.1002/alz70858_103175

**Published:** 2025-12-25

**Authors:** Yaguang Zheng, Katharine Lawrence, Susan Zweig, Richard B. Lipton, Jessica L Zwerling, Linda M. Siminerio, Bei Wu

**Affiliations:** ^1^ New York University, New York, NY, USA; ^2^ NYU Grossman School of Medicine, New York, NY, USA; ^3^ Department of Neurology, Albert Einstein College of Medicine, Bronx, NY, USA; ^4^ Montefiore Medical Center, Bronx, NY, USA; ^5^ Albert Einstein College of Medicine, Bronx, NY, USA; ^6^ Montefiore Einstein, Bronx, NY, USA; ^7^ University of Pittsburgh, Pittsburgh, PA, USA

## Abstract

**Background:**

Mild Alzheimer's disease and related dementia (ADRD) can increase care burden for type 2 diabetes (T2D), which may be particularly prominent for older Chinese Americans. CGM offers a promising solution but is underutilized in this population. This study aimed to examine satisfaction with CGM among older Chinese Americans and their care partners to address gaps in culturally and linguistically tailored diabetes management.

**Method:**

Older Chinese Americans with mild ADRD and T2D (*N* = 11) wore a CGM and real‐time CGM data were shared with their care partners for immediate support of diabetes management. They also received education on how to use CGM data. After 10‐day use, patients and care partners completed the CGM‐Satisfaction Scale individually. The 44‐item scale are scored on a 5‐point Likert (Agree Strongly=1 to Disagree Strongly=5) and comprise two subscales (benefits and hassles). This validated scale has high internal consistency reliability (Cronbach's *α* = .76) and moderate convergent validity (*r* = .29–.34). Higher scores reflect greater overall satisfaction, higher benefits, and lower hassles.

**Result:**

The older Chinese Americans with mild ADRD had a mean age of 74.5 ± 5.2 years, with 63.6% being female, 100% retired, 36.4% at least a college education, and 90.9% family income<$30,000. The care partners were 59.4 ± 20.6 years old, 63.6% female, 54.5% retired, and 54.5% had at least a college education. The primary language of all participants was Chinese. A total of 90.9% of patients and care partners had no prior experience using CGM. The CGM satisfaction total score was (mean ±SD) 4.04±0.46 for patients and 4.03±0.26 for care partners, with scores on the subscale of benefits (patients: 4.21±0.43; care partners: 4.32±0.54) and hassles (patients:4.11±0.57; care partners: 4.09±0.40).

**Conclusion:**

Both older Chinese Americans with mild ADRD and their care partners showed high satisfaction with CGM for diabetes management, with a high level of perceived benefit and a low level of hassles. Findings are informative for the application of CGM in diabetes interventions in older Chinese Americans with mild ADRD and their care partners.

